# Preliminary Results of a Screening Programme for Chlamydia in an Asymptomatic Young Population in Spain

**DOI:** 10.3389/fpubh.2021.615110

**Published:** 2021-02-22

**Authors:** Oriol Yuguero, Jose Manuel Fernández-Armenteros, Álvaro Vilela, Jesús Aramburu, Raquel Laín, Pere Godoy

**Affiliations:** ^1^Institut de Recerca Biomédica de Lleida Fundació Dr. Pifarré (IRBLleida), Lleida, Spain; ^2^Facultat de Medicina, Universitat de Lleida, Lleida, Spain; ^3^Secció de Dermatologia, Hospital Universitari, Lleida, Spain; ^4^Cap Onze de Setembre, Institut Català de la Salut, Lleida, Spain; ^5^Secció Microbiologia, Laboratori Clínic, Hospital Universitari Arnau de VIlanova (HUAV), Lleida, Spain; ^6^Agència de Salut Pública de Catalunya, Lleida, Spain; ^7^Centro Investigación Biomédica en Red (CIBER) de Epidemiología y Salud Pública, Epidemiology and Public Health Networking Biomedical Research Centre (CIBERESP), Madrid, Spain

**Keywords:** sexually transmitted infections, *Chlamydia trachomatis*, screening, prevention, emergencies and health services

## Abstract

**Introduction:** *Chlamydia trachomatis* (CT) infection has increased in recent years, reaching 127 million cases in 2016. Possible complications, especially among women, require intervention for early detection of the infection. The objective of our study was to determine the prevalence of CT infection in a young, sexually active, asymptomatic population.

**Methods:** A cross-sectional study was conducted between December 2017 and 31 December 2018 among young patients aged 18–25 years attending the emergency room for any reason. The presence of CT and other STIs in urine was determined by the Allplex Nucleic Acid Amplification Test (NAAT) with a urine sample. All patients testing positive were followed by the STD unit and tests on all sexual partners/contacts were offered. Moreover, we obtained data about sexual habits and risk factors via a self-reporting questionnaire.

**Results:** One thousand three hundred eight patients were eligible for inclusion of whom 298 consented to participate. Of these, 22/298 (7.4%) were diagnosed with CT. Young people with two or more sexual partners in the last month and those suffering from infection by ureaplasma were at greater risk of infection by CT. Up to 50% of participants do not use barrier methods.

**Conclusion:** The prevalence of infection by CT in the asymptomatic young population is higher than expected according to the recent literature in Spain. The scarce use of barrier methods among this population may be one of the causes of this increase and one of the targets to work on in order to reduce the prevalence of the infection.

## Key Points

- The prevalence of infection by CT in the asymptomatic young population is higher than expected.
- Barrier methods are used in <60% of sexual relations.
- Emergency services can be a good place to implement screening programmes among young people.

## Introduction

The natural history of CT infection has been studied by many researchers (1) but with some study design difficulties due to patient characteristics (2). In 70% of women and 50% of men the infection can be asymptomatic (3). If left untreated, it can persist for months and can cause important long-term sequelae. Genital infection in women can result in pelvic inflammatory disease (PID), which involves a risk of infertility or ectopic pregnancy, notwithstanding complications in pregnancy and postpartum. It can also cause infertility in the male population (4).

According to estimates by the WHO (5), there are at least 127 million CT STI cases worldwide each year (6). In countries with higher gross domestic product, CT infection is the most common STI among young heterosexuals (7, 8). In 2017, Unemo et al. (9) stated that CT infection was the most common bacterial STI and that it causes reproductive complications, especially in women (10). The same review recommends the performance of opportunistic screening in asymptomatic patients or in patients with risk factors for infection. However, the study by Hocking (11) did not reveal the expected results in a population screening programme.

Due to the lack of scientific evidence on what are the best initiatives to control CT infection, the WHO strategy on STIs 2016–2021 (12) recommended investigating the cost effectiveness of screening projects (13). Some countries such as Australia, the United States and Canada have conducted screening programmes (14). The number of projects in Europe has increased in recent years (15), from the first in Norway (16) to others like those in the UK.

Despite the experience of the various programmes, screening in asymptomatic patients is not recommended (9). To date, screening in asymptomatic patients has not proved very cost effective. For example, in the United Kingdom, various screening projects have been conducted comparing the results between asymptomatic patients and other risk groups, without yielding very encouraging results (17).

Other screening programmes have been conducted in asymptomatic patients such as the one carried out in Bangkok in a homosexual population (18), and in Paris (19) on patients attending a specific STI center, where the prevalence was 5.7%.

In Catalonia, according to data from the CEEISAT (20), there has been an increase from 55 cases per 100,000 people in 2016 to 139,90 cases in 2019. A pilot study conducted in our hospital (Lleida, Spain) in 2013 with patients attending for a sexually transmitted infection (STI) yielded a prevalence of infection by CT of 10%, which was higher than expected (21).

The involvement of the Emergency Services in the control of CT infection has been discussed in other initiatives. Currently in Spain, rapid detection tests for CT such as those described by Gaydos et al. (22) are not used. In other countries tests with self-collected samples have been used successfully (23). In 2018, Adamson and Klausner presented the results of the screening programme for CT infection in Australia (24) where emergency services were involved. The aim of our study was to detect the prevalence of CT infection in asymptomatic, sexually active young patients attending the emergency room for reasons unrelated to an STI.

## Methodology

An epidemiological cross-sectional study was conducted on the prevalence of CT infection detected in a screening programme for patients who attended the emergency room between 1 December 2017 and 31 December 2018. The Emergency Department of the Arnau de Vilanova University Hospital receives close to 90,000 patients per year of whom about 7,000 are between 18 and 25 years old.

### Variables

All patients were interviewed with questions based on the CEEISCAT epidemiological survey (25). Subjects' sociodemographic variables (age, sex, country of origin, level of education, and employment status) and epidemiological variables (sexual orientation, number of sexual partners in the last month and year, use of barrier methods and the practice of prostitution), and medical history (previous diagnosis of STIs in the last year and pregnancy) were evaluated. In addition to the detection of CT infection, the same sample was used to detect *Ureaplasma urealyticum, Ureaplasma parvum, Mycoplasma genitalium, Mycoplasma hominis, Trichomonas vaginalis*, and *Neisseria gonorrhoeae*.

The method applied for the diagnosis of CT infection and other STIs was the Allplex™ Seegene® Nucleic Acid Amplification Test (NAAT) STI Essential Assay, in urine for men and women, with 88–95% sensitivity and 95–98% specificity (26) to detect CT infection. This test is recommended by the Catalan Health Department. A urethral sample is regarded as the gold standard specimen type but, due to the nature of this study using asymptomatic emergency room recruitment for practical and acceptability purposes, a urine sample was collected for diagnosis.

The urine samples were sent to the laboratory, stored in the refrigerator at between 4 and 8°C until analyzed within 48 h. The samples were prepared as indicated by the manufacturer (Seegene). DNA from the samples was extracted with EZ1 or QIASymphony (QIAGEN®) equipment. PCR detection of *C. trachomatis* and other microorganisms was performed with the Allplex^TM^ STI-7 V1-1 kit (Seegene®).

The amplification cycle threshold was determined by the CFX96 software using values recommended by the Seegene Allplex testing kit. A valid result required that the sample entered exponential growth with a sigmoid-shaped curve in order to cross the cycle threshold.

In addition, an internal quality control was carried out on the procedure, which if not passed, invalidates the result. Then, extraction must be done again and started again. If the internal control is failed again, it is excluded. In our study there were two samples that need to be repeated, but were finally validated.

Every week, the research team reviewed the participants' results for that period. If the result was negative, no communication was issued to the patient. However, the researchers did inform the patient's Primary care doctor.

Patients with CT positive or result for another STI were referred to the STI Unit at the Arnau de Vilanova University Hospital, Lleida (Spain) where they were administered treatment for the infection, they performed the contact study, and all patients and their sexual contacts had the opportunity to undergo serological tests to detect other sexually transmitted diseases (HIV, Hepatitis B and C and Syphilis).

### Inclusion Criteria

Sexually active patients between 18 and 25 years of age attending the Emergency Room between the dates set out above for reasons not related to a sexually transmitted infection were offered to participate consecutively recruiting 24 h a day, 7 days a week. A person was deemed sexually active if they had had sex in the previous 6 months.

### Exclusion Criteria

Patients who did not agree to participate or who had symptoms that could be caused by CT were excluded.

### Sample Size

In reference to sample size (27), accepting an alpha risk of 0.05 (i.e., 95% confidence) and a beta risk of 0.2 (i.e., a statistical power of 80%) in a two-sided test, a sample size of 1,031 patients is needed to identify a difference ≥0.02% from the expected prevalence of 0.05 described previously (28). No drop-outs are anticipated.

### Statistical Analysis

Qualitative variables were described by absolute frequencies and percentages. For quantitative variables, the median and 25 and 75% percentiles were obtained. The prevalence of CT infection and other STIs were estimated and the 95% confidence interval (CI) was calculated. Whenever application assumptions were held, the Pearson's Chi-square test was used to compare the qualitative variables, and the Mann-Whitney test was used for the quantitative variables. The crude odds ratios (OR) calculated by the Wald method and their 95% CI calculated using normal approximation were also obtained.

A logistic regression model was developed to determine the variables associated with CT infection. The forward selection approach based on the likelihood ratio test was followed to determine the independent variables of the multivariable analysis. Age and sex were included as adjustment variables. Calibration was evaluated using the Hosmer-Lemeshow test, and discrimination with the area under the ROC curve. The adjusted OR are presented, along with the 95% CI. In all analyses an alpha error of 5% was assumed and the R statistical program was used.

## Results

**Figure 1:**
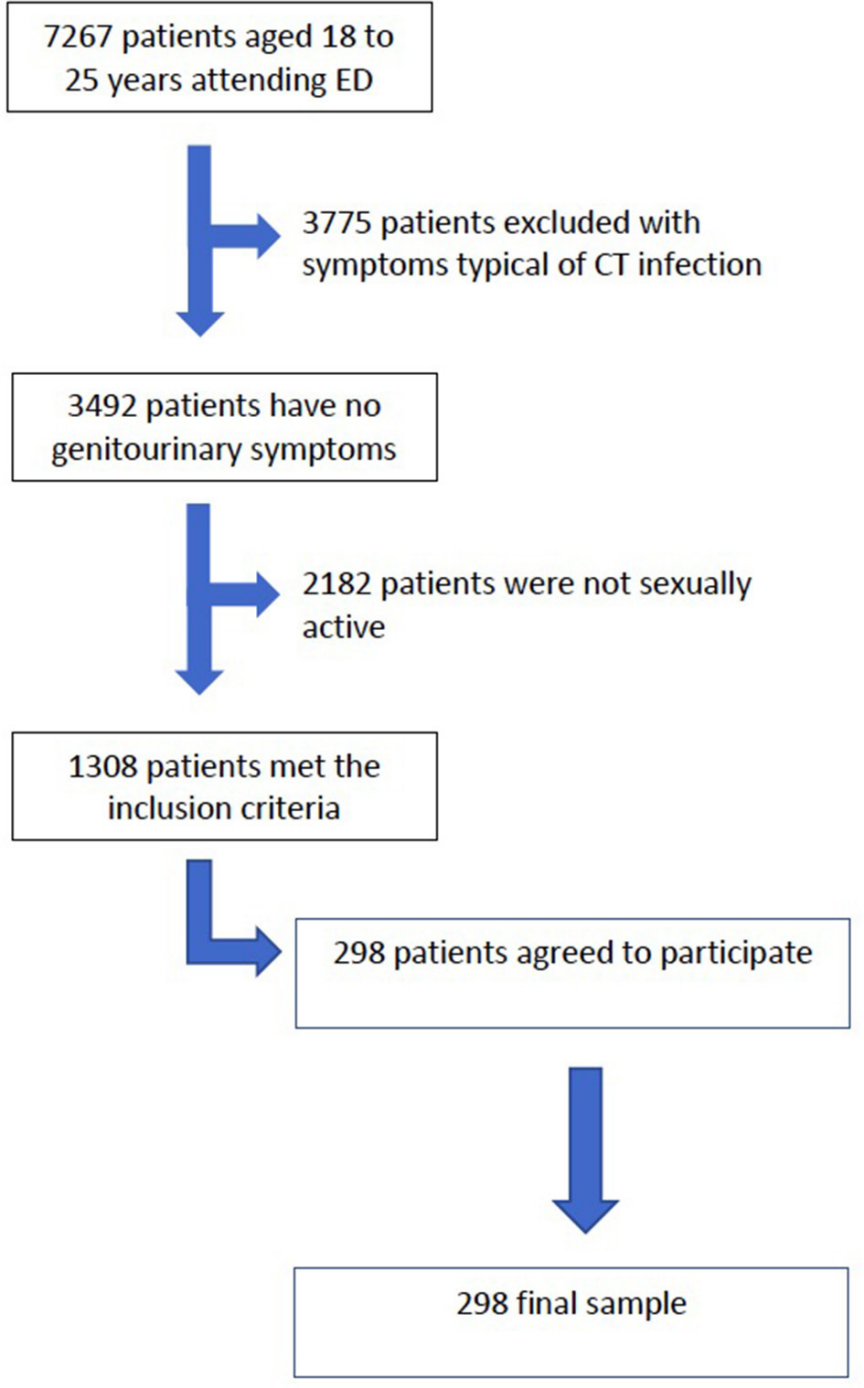
Flowchart of patient inclusion.

During the study period, 7,267 patients aged between 18 and 25 years attended the emergency room. Of these, 3,775 (51.9%) attended with symptoms that could be caused by an infection by CT (abdominal pain, dysuria or genital secretion). Two thousand one hundred eighty-two patients denied being sexually active (30%). Of the 1,308 patients who were eligible to participate in the study, 298 (22.9%) agreed to participate (Figure 1).

54.4% were women and the median age was 22 years. The sociodemographic variables are presented in Table 1. Ninety-three percent of participants reported themselves as being heterosexual, 4.4% bisexual, and the remaining 2.3% homosexual. Just over one-tenth (10.4%) had had two or more partners in the last month, 49.3% had not used a condom in their last sexual intercourse, and 4.4% reported a previous STI (Table 2).

**Table 1:** Sample sociodemographic variables.

	***N* (%)**
	298 (100%)
**Sex**	
Male	136 (45.6%)
Female	162 (54.4%)
**Mean age (P25–P75)**	**22 [20–23.08]**
**Employment**	
Craftspeople and skilled workers in the manufacturing and construction industries (except operators of plant and machinery)	23 (7.72%)
Accounting, administrative, and other office employees	12 (4.03%)
Student	154 (51.7%)
Elementary occupations	8 (2.68%)
Other technical, scientific, and intellectual professionals	7 (2.35%)
Unemployed	30 (10.1%)
Technical, scientific, and intellectual professionals	16 (5.37%)
Qualified agricultural, livestock, forestry, and fisheries employees	25 (8.39%)
Workers in catering, personal, security, and sales services	23 (7.72%)
**Country of origin**	
Spain Rest of Europe Africa	214 (71.8%) 27 (9.06%) 25 (8.39%)
Asia	2 (0.67%)
South America	30 (10.1%)
**Education level**	
No education	6 (2.01%)
Primary	19 (6.38%)
Vocational training	76 (25.5%)
Secondary	82 (27.5%)
University	115 (38.6%)

*Description of the sample included in the study*.

**Table 2:** Sample clinical and epidemiological variables.

	***N* (%)**
	298 (100%)
**Sexual orientation**	
Heterosexual	277 (93.0%)
Homosexual	7 (2.35%)
Bisexual	13 (4.36%)
Unknown	1 (0.34%)
**Stable partner**	
No	117 (39.3%)
Yes	181 (60.7%)
**Number of partners in the last month**	
0–1	267 (89.6%)
2+	31 (10.4%)
**Number of partners in the last year**	
1	188 (63.1%)
2–5	84 (28.2%)
6+	26 (8.72%)
**Exercising prostitution**	
No	295 (99.0%)
Yes	3 (1.01%)
**Routine use of Barrier methods**	
No	124 (41.6%)
Yes	174 (58.4%)
**Condom use at last intercourse**	
No	147 (49.3%)
Yes	151 (50.7%)
**Pregnancy**	
No	291 (97.7%)
Yes	7 (2.35%)
**Previous STI diagnosis?**	
No	285 (95.6%)
Yes	13 (4.36%)
**Within the last year**	
No	297 (99.7%)
Yes	1 (0.34%)

*Clinical and epidemiological characteristics of the patients participating in the study*.

In the screening study, 22 CT-infected patients were detected (7.4%, 95% *CI* = 4.8–11.1%).

An STI was diagnosed in 34 patients (11.4%). In addition to the 22 infected with *CT*, 11 were infected with *U. urealyticum* (3.7%), 2 with *M. genitalium* (0.6%), 1 with *T. vaginalis* (0.3%), and 1 with *N. gonorrhoeae* (0.3%). Three patients were co-infected with CT and Ureaplasma. Only in three patients was serologic screening carried out for hepatitis B, C, syphilis, and HIV. HIV was detected in one of these cases.

CT infection showed no association with age or gender, but manual workers (17.4%), service workers (17.4%), and the unemployed (13.3%) had a higher prevalence of infection than students (3.9%). Patients with secondary school studies also had a higher prevalence of CT than the other groups (Table 3). Approximately 40% of the study participants did not use barrier methods routinely (Table 4).

**Table 3:** Patient demographic variables according to Chlamydia infection.

	**Yes (*N* = 22)**	**No (*N* = 276)**	***P*-Value**	**OR (95% CI)**
**Sex**			0.81	
Male	9 (40.9%)	127 (46.0%)		Ref
Female	13 (59.1%)	149 (54.0%)		1.23 [0.51; 2.97]
**Age**			0.752	
	22.0 [21.0; 23.0]	22.0 [20.0; 24.0]		1.03 [0.85; 1.26]
**Employment**			–	
Craftspeople and skilled workers in the manufacturing and construction industries (except operators of plant and machinery)	4 (18.2%)	19 (6.88%)		
Accounting, administrative and other office employees	1 (4.55%)	11 (3.99%)		
Student	6 (27.3%)	148 (53.6%)		
Elementary occupations	0 (0.00%)	8 (2.90%)		
Other technical, scientific and intellectual professionals	1 (4.55%)	6 (2.17%)		
Unemployed	4 (18.2%)	26 (9.42%)		
Technical, scientific and intellectual professionals	2 (9.09%)	14 (5.07%)		
Qualified agricultural, livestock, forestry and fisheries employees	0 (0.00%)	25 (9.06%)		
Workers in catering, personal, security and sales services	4 (18.2%)	19 (6.88%)		
**Country of origin**			–	
Spain	17 (77.3%)	197 (71.4%)		
Rest of Europe	0 (0.00%)	27 (9.78%)		
Africa	2 (9.09%)	23 (8.33%)		
Asia	1 (4.55%)	1 (0.36%)		
South America	2 (9.09%)	28 (10.1%)		
**Education level**			–	
No education	1 (4.55%)	5 (1.81%)		Ref
Primary	1 (4.55%)	18 (6.52%)		0.28 [0.01; 5.27]
Vocational training	7 (31.8%)	69 (25.0%)		0.51 [0.05; 4.98]
Secondary	8 (36.4%)	74 (26.8%)		0.54 [0.06; 5.22]
University	5 (22.7%)	110 (39.9%)		0.23 [0.02; 2.33]

*Chi square test for sex, and Mann-Whitney test for age. OR obtained univariate logistic regression*.

**Table 4:** Epidemiological and clinical variables of patients infected by Chlamydia.

	**Yes (*N* = 22)**	**No (*N* = 276)**	***P*-value**	**OR (95% CI)**
**Sexual orientation**			–	
Heterosexual	22 (100%)	255 (92.4%)		
Homosexual	0 (0.00%)	7 (2.54%)		
Bisexual	0 (0.00%)	13 (4.71%)		
Unknown	0 (0.00%)	1 (0.36%)		
**Stable partner**			>0.999	
No	9 (40.9%)	108 (39.1%)		Ref
Yes	13 (59.1%)	168 (60.9%)		0.93 [0.38–3.25]
**Number of partners in the last month**			0.064	
0–1	17 (77.3%)	250 (90.6%)		Ref
2+	5 (22.7%)	26 (9.42%)		2.83 [0.96–8.29]
**Number of partners in the last year**			0.326	
1	11 (50.0%)	177 (64.1%)		Ref
2–5	8 (36.4%)	76 (27.5%)		1.69 [0.66; 4.38]
6+	3 (13.6%)	23 (8.33%)		2.10 [0.54; 8.08]
**Exercising prostitution**			–	
No	22 (100%)	273 (98.9%)		
Yes	0 (0.00%)	3 (1.09%)		
**Routine use of Barrier methods**			>0.999	
No	9 (40.9%)	115 (41.7%)		Ref
Yes	13 (59.1%)	161 (58.3%)		1.03 [0.43–2.49]
**Condom use at last intercourse**			0.465	
No	13 (59.1%)	134 (48.6%)		Ref
Yes	9 (40.9%)	142 (51.4%)		0.65 [0.27–1.58]
**Pregnancy**			–	
No	21 (95.5%)	270 (97.8%)		
Yes	1 (4.55%)	6 (2.17%)		
**Previous STI diagnosis?**			–	
No	20 (90.9%)	265 (96.0%)		
Yes	2 (9.09%)	11 (3.99%)		
**Within the last year**			–	
No	22 (100%)	275 (99.6%)		
Yes	0 (0.00%)	1 (0.36%)		

*P-values correspond to the chi-square test. OR obtained univariate logistic regression*.

Twenty-one patients with CT received treatment (95.5%). The one patient who did not receive treatment was due to not attending the Unit or visiting their primary care physician. Focusing on the other STIs, only four patients were treated for Ureaplasma and one for Neisseria. Twelve of the patients with CT identified their contact (54.5%) in the last month. None of the patients reported more than one contact. Nine were diagnosed with and treated for CT. The total number of patients infected with CT was 31.

Logistic regression analysis showed that patients who had had two or more partners in the last month [OR 3.8; 95% CI (1.2–12.1)] and were infected with Ureaplasma [OR 6.8; 95% CI (1.6–29.2)] had a higher risk of CT infection.

## Discussion

The prevalence of *Chlamydia trachomatis* infection in young asymptomatic patients is 7.4% in the screened patients. This figure is higher than that previously described in our environment, since prevalence had been estimated at 5% (29). In 2010 a similar study was conducted in Spain that showed a prevalence of 4% (30).

This is the first screening study for infection by CT in young asymptomatic subjects in Catalonia, Spain. The study results are preliminary, although we have considered that they are sufficiently important to be communicated. We believe that the population attending out-of-hours healthcare services was a good choice since it is difficult to contact young patients as they tend to visit the emergency room for acute problems or accidents (31).

The mean age of our sample was 22 years and there was a predominance of women (54.5%). However, there were no associations with gender, age, sexual orientation or country of origin.

There was an association with the number of sexual partners in the last month: the greater the number of sexual partners in the last month, the higher the risk of CT infection. This was already described in 1992 by Joffe et al. (32) and recently by Lopez-de-Munain in a recent study (33) which also revealed a higher risk of infection in a cohort of patients who attended an STI unit and reported two or more sexual partners in the last month.

The study allowed us to detect 31 cases of CT in asymptomatic patients (22 cases via Emergency Room screening and nine via contact tracing of these positive cases), thus preventing the spread of the disease and the subsequent complications that may arise from asymptomatic infection. Also, in the subsequent study, we detected one case of HIV in an asymptomatic patient. Probably, if HIV serology had been performed on a larger number of patients, more cases would have been detected. It was disappointing that many patients have not undergone serological tests despite their positive results for other STIs. However, serologies can be a marker of past exposure rather than current infection, especially for viral infections like HIV or HBV, but recent recommendations from Spanish guidelines (34) recommend testing for HIV in all patients with different STIs to detect hidden infections.

The co-infection of CT with *U. parvum* is notable. This association was also described in other studies. Kim et al. (35), Ndeikoundam Ngangro et al. (36), and Yamazaki (37) suggest that the presence of Ureaplasma has a significant effect on the presence of *C. trachomatis* in the genital tract. Berçot et al. (38) go further and relate this co-infection with the establishment of chronic *C. trachomatis* infection. We believe the possibility of using tests that detect various infections in screening programmes may be in order to achieve greater patient information, although there is a need to perform cost-benefit studies and discard overdiagnosis and overtreatment before recommending their use.

One study (39) showed that even in asymptomatic women there is a high prevalence of STIs whether they belong to a high-risk group or not, as our results confirm.

We also believe it is relevant that ~40% of participants do not use barrier methods regularly and did not use a condom upon their last sexual intercourse. This is remarkable given the multitude of awareness campaigns that exist and the little impact they have on young people. This situation has been described previously, specifically in campaigns on STIs (40). Due to their apparent lack of effectiveness, strategies to promote condom use and improve their impact on young people should be reviewed.

To date, for reasons of cost-effectiveness, in Spain, screening programmes for CT had only been conducted in patients with risk factors, such as patients attending STI clinics (21) or pregnant women (41). Through an opportunistic case-finding strategy, the project took advantage of visits by young sexually active patients to an emergency room to reach one of the main risk groups which, being asymptomatic, is very unlikely to attend the physician's office.

We believe that detecting CT infection in 7.4% of young people highlights that prevalence could increase in the following years and justifies planning strategies to detect infection in young patients since current education and awareness strategies show little impact. The benefit of receiving treatment and avoiding complications and sequelae is sufficiently important to consider implementing a nationwide strategy.

The main limitation of our study is the current sample size. We have achieved a response from 23% of the participants, hence the size of our sample is small, and this could lead to sample bias. However, the screening programme continues. Moreover, another limitation is that younger patients are sometimes accompanied in the emergency room by relatives in whose presence these patients do not admit to the practice of sexual relations. Also, the out-of-hours healthcare services user population may not be representative of all young people.

Our study provides evidence of the importance of a screening programme, but also the importance of campaigns to raise awareness in the use of barrier methods, since the number of sexual partners is still the main factor conditioning CT infection. Finally, we think that these results encourage us to continue with the study and to promote screening programmes among the general population.

## Data Availability Statement

The raw data supporting the conclusions of this article will be made available by the authors, without undue reservation.

## Ethics Statement

The study was approved by the Ethics Committee of the Lleida Biomedical Research Institute (IRBLLEIDA) with reference CEIC-1844) and all the research has been conducted following the statements of the Declaration of Helsinki on Biomedical Research. All patients signed a form of informed consent for their inclusion in the study and patient data were processed according to the Spanish Organic Law 3/2018 on Data Protection.

## Author Contributions

OY and PG contributed to the conception and design of the work. JF-A, RL, and ÁV participated in data acquisition and interpretation. JA drafted the work and revised it critically. All the authors gave their final approval of the version for publication.

## Conflict of Interest

The authors declare that the research was conducted in the absence of any commercial or financial relationships that could be construed as a potential conflict of interest.
